# Molecular Profiling of Circulating Tumour Cells and Circulating Tumour DNA: Complementary Insights from a Single Blood Sample Utilising the Parsortix^®^ System

**DOI:** 10.3390/cimb46010050

**Published:** 2024-01-17

**Authors:** Gabrielle Wishart, Amy Templeman, Francesca Hendry, Karen Miller, Anne-Sophie Pailhes-Jimenez

**Affiliations:** ANGLE plc, Guildford GU2 7QB, UK; a.templeman@angleplc.com (A.T.); f.hendry@angleplc.com (F.H.); k.miller@angleplc.com (K.M.); a.pailhes-jimenez@angleplc.com (A.-S.P.-J.)

**Keywords:** blood, cancer, liquid biopsy, circulating tumor cells, circulating tumor DNA, cell-free DNA, microfluidic devices, neoplastic cells

## Abstract

The study of molecular drivers of cancer is an area of rapid growth and has led to the development of targeted treatments, significantly improving patient outcomes in many cancer types. The identification of actionable mutations informing targeted treatment strategies are now considered essential to the management of cancer. Traditionally, this information has been obtained through biomarker assessment of a tissue biopsy which is costly and can be associated with clinical complications and adverse events. In the last decade, blood-based liquid biopsy has emerged as a minimally invasive, fast, and cost-effective alternative, which is better suited to the requirement for longitudinal monitoring. Liquid biopsies allow for the concurrent study of multiple analytes, such as circulating tumour cells (CTCs) and circulating tumour DNA (ctDNA), from a single blood sample. Although ctDNA assays are commercially more advanced, there is an increasing awareness of the clinical significance of the transcriptome and proteome which can be analysed using CTCs. Herein, we review the literature in which the microfluidic, label-free Parsortix^®^ system is utilised for CTC capture, harvest and analysis, alongside the analysis of ctDNA from a single blood sample. This detailed summary of the literature demonstrates how these two analytes can provide complementary disease information.

## 1. Background

### 1.1. Tumour Burden and Heterogeneity

The global burden of cancer challenges human health and the economy and was responsible for nearly 10 million deaths in 2020 [1] (accessed on 18 September 2023). Rising prevalence and incidence rates call for effective diagnostics and treatment selection strategies. Furthermore, the dynamic landscape of cancer demands continuous up-to-date and accurate monitoring methods for effective patient care [2]. This intertumoral and intratumoral heterogeneity of cancer as a basis for tumour evolution, treatment resistance and subsequent treatment failure, is an area of growing understanding [3]. Recent advances in high-throughput, relatively low-cost sequencing techniques (for example, next generation sequencing (NGS)) have shed light on molecular drivers of cancer, actionable mutations and the continuous process of clonal evolution from selective pressure of cancer therapies [4]. As such, it is widely accepted that personalised or precision medicine will optimise response to cancer therapy and improve quality of life for the patient.

The standard of care for evaluating patient specific biomarkers, mutations, and genetic signatures for the appropriate selection of targeted cancer treatments is to conduct a tissue biopsy. Despite being the most widely used technique, tissue biopsies have numerous disadvantages such as being invasive, costly, failing to capture tumour heterogeneity, and being harmful to the patient [5]. Moreover, tissue biopsies are rarely suitable for longitudinal monitoring where the patient is too unwell and where the patient’s primary tumour has been excised, or metastasised to two or more sites [6]. Liquid biopsy techniques are advancing to provide a less invasive, safer, less costly alternative that provides results faster than tissue biopsies [7]. Furthermore, this technique is better suited for longitudinal disease monitoring and captures heterogeneity and the clonal evolution responsible for treatment failure and drug resistance [7].

### 1.2. Liquid Biopsy

**Figure 1:**
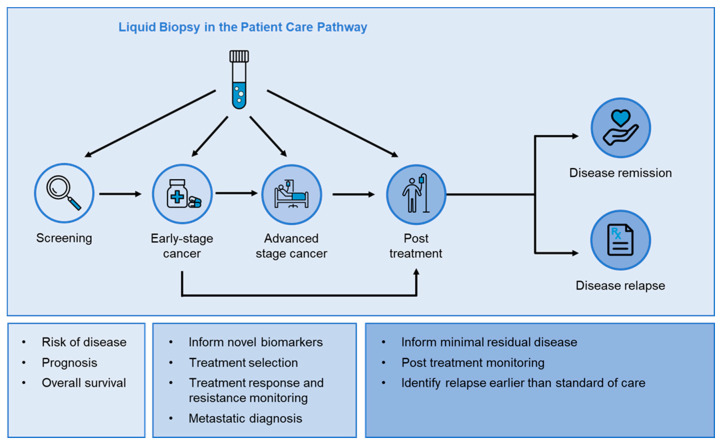
Clinical utility of liquid biopsies across the patient care pathway. Liquid biopsies are minimally invasive tools used (1) in patient screening to predict risk of disease, prognosis, and overall survival; (2) in early-stage cancer to inform targeted therapies for first-line treatment, identify novel biomarkers, and to monitor treatment response and to provide an early predictor of treatment resistance; (3) at disease progression in advanced stage cancer to confirm metastatic diagnosis, inform targeted treatment selection, monitor treatment response and treatment resistance, and identify new drug targets as the tumour evolves (clonal evolution); (4) post treatment to identify minimal residual disease, monitor the patient during remission, and identify risk of relapse. Liquid biopsies allow the analysis of different blood-based analytes including circulating tumour cells (CTCs) and cell-free DNA (cfDNA). The latter provides genomic information from fragmented DNA, whereas CTCs are whole cells providing not only genomic, but transcriptomic and proteomic information for a more inclusive view of the current state of tumour mutations and biomarkers towards personalised therapy.

A liquid biopsy is a minimally invasive test performed using bodily fluids, such as blood, and it has received growing clinical interest for its applications in personalised medicine [8]. Blood-based liquid biopsies allow for the analysis of circulating tumour cells (CTCs), cell-free DNA (cfDNA), circulating tumour DNA (ctDNA), or other plasma components, such as cell-free RNA, proteins, and exosomes, to provide clinically relevant and actionable information. More specifically, liquid biopsies have shown significant relevance across the cancer care pathway, informing cancer diagnosis, prognosis, treatment selection, the monitoring of disease evolution, and disease relapse [7,8] (Figure 1). The advancement of sequencing technology is fuelling a revolution in liquid biopsy analysis, providing genomic and transcriptomic characterisation for personalised therapy selection [9]. Liquid biopsies are also emerging as valuable tools for drug discovery and development having applications as prognostic and pharmacodynamic biomarkers, with several consortiums founded to analyse, implement, and develop standards for liquid biopsy in clinical trials and drug development. These include Friends of Cancer research ctMONiTR, the International Liquid Biopsy Standardization Alliance (ILSA), the Blood profiling Atlas in Cancer (BloodPAC) Consortium and Cancer ID.

### 1.3. Circulating Tumour DNA

Cell-free DNA (cfDNA) consists of DNA fragments found in the body fluids of healthy and non-healthy patients and is thought to be derived from cellular breakdown mechanisms [10]. cfDNA circulates in fragments typically ranging in size from 120 to 140 base pairs [10]. In cancer patients, circulating tumour DNA (ctDNA) accounts for a very low percentage (0.01–1%) of total cfDNA and is specifically derived from the tumour [11]. The origin of ctDNA is not fully understood, but is believed to be from apoptotic cells, necrotic cells or to enter the bloodstream via active secretion [8,12,13]. The profiling of ctDNA has received attention for early diagnosis, treatment selection, the identification of resistance mechanisms and detection of post-surgical minimal residual disease in numerous cancer types [4,12,14]. The analysis of ctDNA provides a simple method of obtaining genetic information; however, this is limited to point mutations, structural rearrangements, copy number variants (CNVs) and changes in DNA methylation [4]. It has been reported in the literature that genetic signatures in ctDNA can be derived from the major clone in a tumour and thus, subclonal signatures may be missed when studying this analyte [5]. However, ctDNA and cfDNA are the most established liquid biopsy analytes in the oncology market with five FDA-approved companion diagnostics for targeted treatment selection and residual disease monitoring [15] (accessed on 1 December 2023). For example, the cobas epidermal growth factor receptor (EGFR) mutation test V2 (Roche) to detect EGFR mutations (Exon 19 deletion or exon 21 L858R substitution mutation) in non-small-cell lung cancer (NSCLC) patients for treatment with Tagrisso (osimertinib) and Tarceva (erlotinib) [16,17] (accessed on 9 January 2024).

### 1.4. Circulating Tumour Cells

CTCs are whole cells released by a tumour into the bloodstream and are responsible for metastatic seeding [18,19]. CTC enumeration provides robust prognostic information; increased CTC presence correlates to metastatic burden, with a strong association with overall survival in numerous cancer types [4]. Beyond CTC enumeration, CTCs can provide functional genomic, transcriptomic and proteomic information, providing accurate tumour phenotypic information at the time of sampling [2,3,8]. This presents a unique real-time window into clinically relevant information towards personalised treatment. This analyte has been reported to reflect high levels of tumour heterogeneity [3] and represent clonal evolution that may be responsible for treatment failure and drug resistance [2,8,19]. As such, CTCs are suitable for treatment selection [2], real-time longitudinal disease monitoring, treatment monitoring, and relapse monitoring [5]. Furthermore, harvesting CTCs from blood facilitates research into the complex landscape of cancer including CTC clustering, cellular invasion, and metastasis [4] and is suitable for in vitro/in vivo culture research [20]. CTCs are an area of growing interest across multiple cancer types [2,8] and are emerging as a tool to address challenges of the complex landscape of heterogeneity in the clinic. As such, there is a demand for enrichment technologies that are able to successfully isolate rare CTCs from whole blood. Numerous CTC enrichment technologies are emerging based on a cell’s physical properties, biological properties and a combination of the two [19,21]. These include membrane microfilters, microfluidic technologies, non-microfluidic technologies, positive selection by tumour marker technologies, and negative selection by non-tumour marker technologies [19,21]. Technologies face challenges in isolating CTCs given their rarity, their phenotype, size heterogeneity and the need for downstream analysis [22,23]. Often, there is a reported trade-off between CTC recovery and sample purity [22]. Microfluidic CTC isolation technologies have received attention for high throughput, sensitivity, low sample consumption and cost [23].

Currently there are only two FDA-cleared medical devices for the enrichment of CTCs. These include the CellSearch^®^ Circulating Tumor Cell (CTC) Test (Menarini-Silicon Biosystems, Huntingdon Valley, PA, USA): *for the enumeration of CTCs of epithelial origin for the monitoring of prognostic information of patients with metastatic breast, colorectal, or prostate cancer,* and the Parsortix^®^ PC1 System (ANGLE plc, Guildford, UK): *for the capture and harvest of CTCs from the blood of metastatic breast cancer (MBC) patients for subsequent, user-validated analysis*. CellSearch^®^ isolates and detects CTCs of epithelial origin via an immunoaffinity-based enrichment method. However, CTCs can exist in three subtypes including epithelial, mesenchymal, and epithelial/mesenchymal CTCs, thus cells undergoing or having undergone epithelial to mesenchymal transition (EMT) (a process that increases metastatic properties of cancer cells, enhancing cellular migration and invasion [24]) may be missed by such enrichment technologies. The Parsortix^®^ system overcomes this issue with epitope-independent CTC capture, isolating epithelial, mesenchymal, and epithelial/mesenchymal CTCs. Furthermore, the subsequent downstream analysis of CTCs provides a wealth of information as compared to CTC enumeration.

### 1.5. The Parsortix^®^ System

The Parsortix^®^ system is a liquid biopsy platform that uses a patented microfluidic technology enabling label-free (epitope-independent) capture of all CTC phenotypes based on cell size and deformability, allowing for CTC enumeration and subsequent downstream analysis [25]. More specifically, the Parsortix^®^ system can facilitate the capture [25] of CTCs, as well as the harvest of CTCs for subsequent downstream analysis methods, [26] including individual gene expression analysis (messenger RNA [mRNA]) and protein evaluation (e.g., cytological/immunofluorescent [IF] staining) [27,28,29], the evaluation of DNA aberrations [30], and whole genomic [31] and transcriptomic sequencing [32], amongst others. These subsequent downstream methods have been utilised in the literature as tools for studying CTCs and the tumour microenvironment [33,34], identifying clinically actionable targets [30] towards therapeutic screening [31]/patient cohort selection and personalised treatment, resistance profiling [35], and drug discovery [33] and development [14].

These applications and techniques used in tandem with the Parsortix^®^ system are explored in 92 peer reviewed publications from 38 independent study centres across 18 cancer types [36] (accessed on 28 September 2023). The Parsortix^®^ PC1 Clinical System’s analytical performance [37] and multi-centre clinical performance [26] has been demonstrated to capture and harvest CTCs, and provide specific, user-validated downstream analysis in MBC. Moreover, the Parsortix^®^ system is currently under evaluation in clinical trials to investigate therapeutic influence on CTC clusters [38], the role of sleep in the spread of CTCs in lung cancer patients [39], and to investigate the intestinal polyp secretion of tumour cells and circulating factors [40].

## 2. Molecular Advances: The Omics Revolution

In the last 20 years, there have been exponential advances in the understanding and application of molecular analysis and computational tools as genomic sequencing has become well-established and affordable. More recently, it is understood that studying the genome provides a basis of information that is just the beginning of a complex biological landscape and that we are able to look beyond the genome [41]. Genomic information can be supplemented with transcriptomic and proteomic data for closer evaluation of tumour phenotype towards more accurate, real-time information for personalised treatment (the study of multi-omics). Genomic analysis provides information on past mutations acquired during the evolutionary history of the tumour, whereas transcriptomic analysis provides a window into epigenetic influence on gene expression and thus the current state of the tumour [42]. This interplay between the genome and the transcriptome is relevant for identifying up-to-date and accurate treatment options [42]. The importance of studying the transcriptome has been demonstrated in real-world clinical data, in which tissue-derived RNA sequencing discovered more clinically actionable targets than DNA sequencing alone, increasing the number of patients eligible for matched therapies by 24% [43]. Similarly, other research has shown that utilising transcriptomics can increase the number of patients administered for matched therapy [44]. Moreover, it is predicted that by harnessing NGS tools and the nature of transcriptomics, it is possible to head towards a new era of personalised medicine, something which is recognised by the National Institute of Health [45]. As such, we are entering an omics revolution that aims to progress personalised medicine [41]. Furthermore, this evolution of molecular technology has necessitated the concurrent development and application of artificial intelligence and machine learning for the integration of big data into the clinic [46].

These molecular advances are fuelling liquid biopsy analysis [9]. Advances in digital polymerase chain reaction (dPCR) and sequencing technologies are facilitating low-cost, rapid analysis, with limited starting material to provide clinically relevant multi-omic information [47]. Notably, the application of NGS technology is enabling the identification of druggable targets, clonal selection, and metastatic information from liquid biopsy analytes as real-time tools [30]. There are 20 peer reviewed publications that utilise the Parsortix^®^ system and NGS technology to study the genome or transcriptome. More specifically, eight of these publications perform bulk harvest NGS analysis, and 12 study the use of single-cell analysis. As such, the ability of CTCs to provide both genomic and transcriptomic information in addition to genomic information from ctDNA as dual analytes from the same patient sample is an exciting prospect. Currently, there is no single device or companion diagnostic approved for the combined analysis of CTCs and ctDNA or multi-analyte analysis from a single blood sample, but dual analysis is emerging in the literature.

## 3. Complementary Insights

In the literature, the enumeration of CTCs and analysis of ctDNA in tandem have previously informed prognosis across various cancer types [48,49]. Rapid advances in CTC isolation technologies and the omics revolution have enabled the molecular analysis of both CTCs and ctDNA as a minimally invasive approach to define tumour heterogeneity and clonal evolution to study metastasis [4]. As this information is imperative for treatment success, CTCs and ctDNA have been described as cornerstones of liquid biopsy diagnosis, paving the way for new diagnostic opportunities [8]. Until recently, the analysis of CTCs and ctDNA have been referred to in the literature as competing sources of information [50]; however, there has been a shift in understanding that the two analytes can provide complementary insights [3,8], expanding the amount of clinically actionable information to inform the patient care pathway.

**Figure 2:**
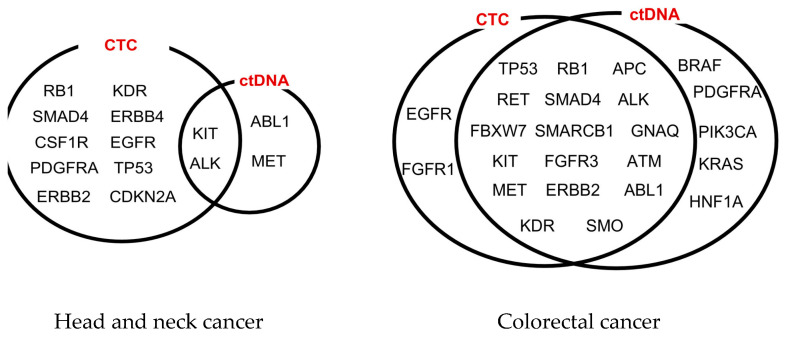
CTC and ctDNA analysis of genomic alterations in head and neck and colorectal cancer patients. Figure reproduced from Onidani et al. (2019) [52].

Aoki et al. (2020) describe the dual analysis of these analytes to increase genomic mutation profiling sensitivity without decreasing specificity [51] and alludes to the unique ability of CTCs to provide novel genomic, transcriptomic, proteomic, metabolomic, and secretomic information in the future. Onidani et al. (2019) conducted NGS research into the genomic profiles of CTCs and ctDNA via the targeted sequencing of 37 head and neck or gastrointestinal cancer patients [52]. They reported that in both cancer types, patients identified with both concordant and discordant clinically actionable information within CTCs and ctDNA (Figure 2). For example, in some head and neck cancer patients, mutations in *ALK* and *KIT* were present in both analytes, whereas mutations in *TP53* and *SMAD4* were exclusive to CTCs and mutations in *MET* were exclusive to ctDNA. Similarly, in colorectal cancer patients, mutations in *TP53* and *SMAD4* were present in both analytes, whereas *EGFR* mutations were exclusive to CTCs, and *BRAF*, *KRAS* and *PIK3CA* mutations were exclusive to ctDNA. The authors state that CTCs and ctDNA exhibited genetic heterogeneity and that dual analysis is more informative than using one analyte alone, outlining the relevance of this tool for real-time monitoring of disease progression, treatment selection and personalised care [52]. Similarly, Manier et al., (2018), performed research into 28 multiple myeloma patients to report that whole exome sequencing (WES) revealed mutations exclusive to either CTCs or cfDNA. These analytes presented different genetic profiles for the cross-evaluation of mutations, and the research infers that this complementary information provided a comprehensive profile of clonal heterogeneity in multiple myeloma [53]. This research also reports that in specific cases, the actionable biomarker *TP53* was mutated in both CTCs and ctDNA but not in primary tissue samples, highlighting the benefit of liquid biopsy [53].

Kong et al. (2020) performed CTC and ctDNA mutation profiling via qPCR and Sanger sequencing in 16 lung adenocarcinoma and 21 breast ductal carcinoma patients. This research reported that higher degrees of genomic heterogeneity were present in CTCs as compared to ctDNA. More specifically, in some breast cancer patients, clinically actionable mutations such as *JAK3*, *BRAF* or *MTOR* amplifications were present at specific timepoints in CTC analysis but absent in matched ctDNA. The authors hypothesise that the difference may stem from the origin of the analytes; CTCs may have evolved and survived treatment whereas the ctDNA may be presenting genetic information of apoptotic tumour cells. Furthermore, when analysed together, CTCs and ctDNA displayed higher degrees of concordance with the metastatic tumour as compared to the primary tumour, representing clonal evolution. In detail, this evidence indicates that dual analysis detected evolving signatures during the progression of disease and throughout treatment, highlighting the potential for use as treatment guides in personalised therapy [5]. Other research articles support the findings that dual analysis of these analytes provides complementary profiling information [54,55]. Some authors state that single-cell profiling of CTCs allows tumour heterogeneity insights beyond that of ctDNA alone, and that the addition of CTCs to the study of cfDNA is clinically relevant for monitoring clonal evolution and relapse [3].

Keup et al., report on a project named ELIMA (‘evaluation of multiple liquid biopsy analytes in metastatic breast cancer patients all from one blood sample’) in which they published a series of investigations assessing the mutation profiles in three or more blood-based analytes. Keup et al. (2021) evaluated CTC mRNA, extracellular vesicle (EV) mRNA and cfDNA profiles in 27 hormone receptor positive, HER2 negative MBC patients, reporting that the largest and most diverse number of overexpression signals occurred within CTCs [56]. The authors state that this diversity mirrors spatial tumour heterogeneity, a leading cause of treatment failure. Moreover, EV signals fluctuated greatly showing that temporal heterogeneity and cfDNA provided a source for actionable variants. Thus, all three analytes were complementary and together provided longitudinal, multiparametric information to capture heterogeneity and tumour evolution [56]. In a similar ELIMA study, Keup et al. (2021) evaluated CTC mRNA, CTC gDNA, EV mRNA and cfDNA from 26 hormone receptor positive, HER2 negative MBC patients via qPCR, finding that a combination of two analytes resulted in 81–92% of patients presenting with actionable signals, a combination of three resulted in 92–96%, and all four resulted in 96% of patients presenting with an actionable mutation signal [57]. Thus, these analytes are complementary as opposed to competitive, and enable genomic and transcriptomic disease characterisation towards more effective personalised medicine.

In the literature, the number of articles published including both CTCs and cfDNA/ctDNA blood analytes is low in comparison to the analytes studied alone. The rapid evolution of this research field may influence this in the future. Currently, clinical trials undertaking the dual assessment of CTCs and ctDNA are underway in a number of cancer types to assess patterns in diagnosis [58], to monitor biomarker response to treatment [59,60], and to test if dual analysis is more sensitive than standard parameters and imaging for disease monitoring [61]. It is suggested that the current limited access to both CTC enrichment platforms and ctDNA sequencing platforms in the same laboratory is responsible for the rarity of dual analysis research articles [5]. Moreover, it is reported that in some studies, the dual analysis of CTCs and ctDNA has taken place, but only epithelial CTCs have been isolated, thus mesenchymal or EMT phenotypes were missing. Furthermore, some studies have only focused on a single mutation and therefore lack comprehensive profiling, and others study CTCs and ctDNA from different blood samples, failing to account for inter-sample heterogeneity [5]. The Parsortix^®^ system can address these issues as a label-free tool for the isolation and harvest of CTCs, facilitating the analysis of CTCs in conjunction with ctDNA from the same blood sample. Herein, we review the literature in which the Parsortix^®^ system has been utilised for this dual analysis.

## 4. The Parsortix^®^ System and Dual Analysis

The Parsortix^®^ system has been used in studies investigating complementary information from CTCs and ctDNA in NSCLC [62,63,64,65,66], triple negative breast cancer (TNBC) [67], head and neck cancer, colorectal cancer, and melanoma [68]. These studies include dual analysis towards the evaluation of prognosis [62,68], biomarker treatment selection [62,67] and to inform treatment resistance [64,66] and relapse [62,63] faster than the standard of care [69]. This showcases the clinical utility of liquid biopsy dual analysis throughout the patient care pathway. A selection of these peer reviewed publications is discussed below and listed in Table 1.

**Figure 3:**
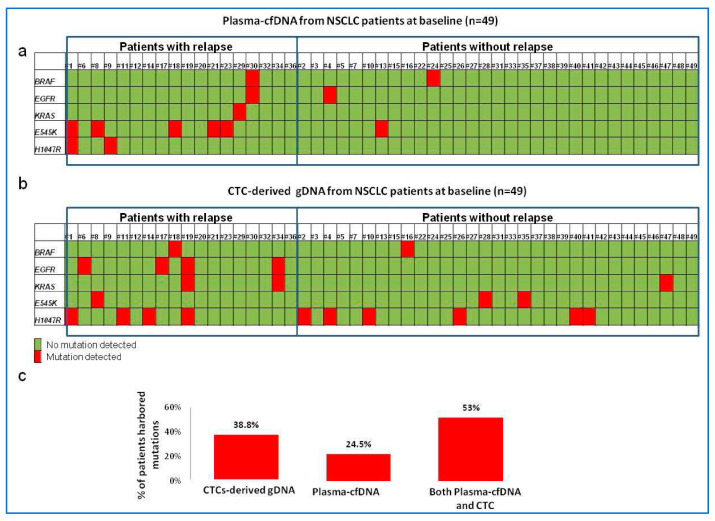
Mutation status in four therapeutically relevant genes (*BRAF*, *KRAS*, *EGFR*, and *PIK3CA: E545K* and *H1045R*) from 49 early-stage NSCLC patients in (**a**) CTC-derived DNA and (**b**) plasma ctDNA and (**c**) the percentage of patient mutations from CTC-derived DNA alone, plasma ctDNA alone or analysed in combination. Red represents mutation. Green represents wildtype. Figure reproduced from Markou, A.N. et al. (2023) [62].

Markou et al. (2023) investigated Parsortix^®^-enriched CTCs and also cfDNA for hotspot mutations in four therapeutically relevant genes (*BRAF*, *KRAS*, *EGFR*, and *PIK3CA: E545K* and *H1045R*) from 49 early-stage NSCLC patients via droplet digital PCR (ddPCR) to find complementary genomic information from the same blood sample. The prevalence of the mutations tested was higher in CTCs as compared to cfDNA (38.8% and 24.5%, respectively), and high heterogeneity was present both within and between the analytes. The combined analyses of CTCs and cfDNA increased the percentage of patients identified with actionable mutations to 53%, highlighting the benefit of dual analysis (Figure 3). Moreover, this research showed that the incidence of progression and relapse was higher when at least one mutation was detected in either sample, as compared to no detectable mutation, revealing important stratification factors for early-stage NSCLC. As such, these samples provided diverse genomic information regarding the prognosis and treatment of NSCLC [62]. 

The same research group investigated the prognostic value of DNA methylation detection in five gene promoters (*APC*, *RASSFIA1*, *FOXA1*, *SLFN11*, *SHOX2*) in early-stage NSCLC patients via real-time methylation-specific PCR (MSP). Beyond DNA mutations, epigenetic changes in methylation patterns can influence tumour suppressor gene expression and can be identified as an early event in tumorigenesis. This study reports differences in DNA methylation patterns in CTCs, cfDNA and the primary tumour, as well as a higher incidence of relapse when at least one methylated gene promoter was detected in CTCs or cfDNA, highlighting the complementary nature and prognostic benefit to dual analyte analysis. The authors state that the dual analysis of CTCs and cfDNA allow for real-time monitoring of tumour evolution [63]. Similarly, Ntzifa et al. (2021) investigated the DNA methylation patterns of nine genes (*RASSF1A*, *RASSF10*, *APC*, *WIF-1*, *BRMS1*, *SLFN11*, *RARβ*, *SHISA3*, and *FOXA1*) in NSCLC patients during osimertinib treatment to find complementary information in CTCs and cfDNA. This research reported that the presence of at least one gene methylation pattern was associated with progressive disease and identified methylation as a potential resistance mechanism [64].

Mondelo-Macía et al. (2020) reported the successful detection of MET (hepatocyte growth factor receptor) expression in CTCs (via immunofluorescence) and amplification in cfDNA (via ddPCR) in a variety of cancer types (lung, colon, prostate, melanoma, breast, and gastric cancer patients) towards the characterisation of tumours and for the detection of treatment resistance [68]. More specifically, a correlation between cfDNA concentration and *MET* copy number was determined. Furthermore, an association between CTCs that were MET positive and poor survival in head and neck cancer patients was reported, an association not observed for MET amplification determined by cfDNA analysis [68]. This research highlights the potential for both CTC and cfDNA analysis as useful tools for characterising tumours and guiding personalised treatment upon detection of treatment resistance, through longitudinal monitoring.

**Figure 4:**
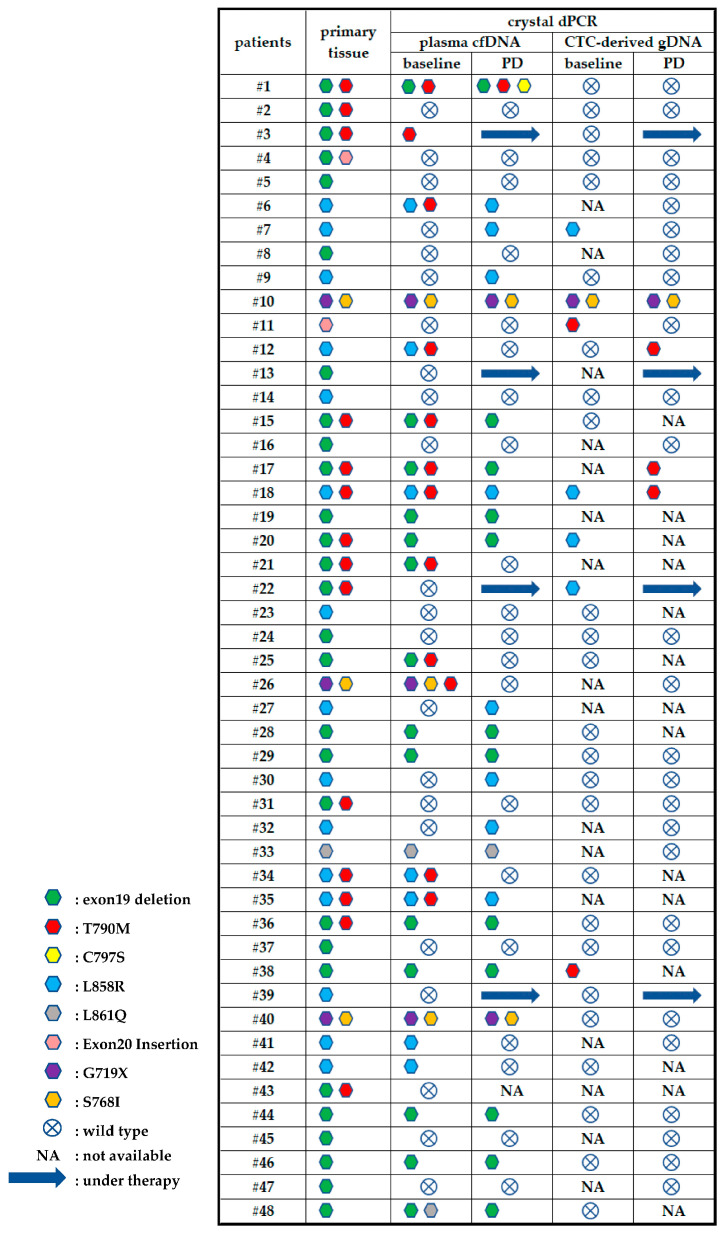
EGFR mutation comparison in primary tissue, plasma cfDNA and CTC-derived gDNA. Figure reproduced from Ntzifa, A. et al. (2021) [66].

In a study by Ntzifa et al. (2021), the presence of EGFR mutations in tissue, cfDNA and CTCs in NSCLC patients undergoing osimertinib therapy was determined using Crystal Digital PCR^TM^ and subsequently compared (Figure 4). Of note, two patients (#11 and #38) with a T790M mutation (a mutation associated with resistance to EGFR inhibitors) detected in CTCs at the baseline but not in cfDNA or tissue had significantly lower progression-free survival. Moreover, the presence of the T790M mutation was detected in CTCs from three patients (#12, #17 and #18) at disease progression, which was absent at this time in cfDNA. The authors reported that this may be indicative of tumour heterogeneity and could also be predictive of resistance mechanisms occurring under selective treatment pressure. The authors conclude that analysis of EGFR mutations in both CTCs and cfDNA could be more informative for treatment monitoring in these patients [66].

Ortolan et al. (2021) evaluated CTCs and ctDNA in 42 patients with early-stage TNBC, via ddPCR and NGS. The authors state that ctDNA presence was indicative of relapse events and may help stratify patients suitable for intensification or alterative treatment post therapy to prevent metastasis development. Furthermore, CTCs analysed at disease progression revealed unique genetic abnormalities such as gain/loss of chromosome 10 and 21q. Network analysis of these altered regions identified actionable pathways including PI3K/Akt, erbB, Raf, platinum-resistance signalling, and regulation of immune response. This research states that CTCs were non-epithelial in most cases, as such they would not have been detected by epithelial dependent CTC enrichment technologies. Overall, the research team endorsed blood-based genomic analyses to utilise ctDNA as a tool for treatment response monitoring and CTCs as a tool to explore druggable targets in disease progression in TNBC patients [67].

In a study by Gorges et al. (2019), CTCs and ctDNA samples from 84 melanoma patients underwent the analysis of 61 clinically relevant variants across 13 genes including *BRAF*, *NRAS* and *MAP2K1*. The study reported that ctDNA and CTCs provided complementary information, indicated relapse prior to standard of care imaging, and were more accurate than the current melanoma staging system and biomarkers in some patients. More specifically, in one case, CTCs presented with BRAFV600E and EGFRI491M mutations at patient relapse, guiding targeted therapy; however, neither ctDNA, LDH (lactate dehydrogenase), or S100 (a melanoma marker gene) levels were elevated at this time. This research concludes that CTCs and ctDNA together provide real-time, complementary information on the mutational status of RNA and protein expression, with clinical significance for melanoma patients [69]. Aya-Bonilla, et al. (2020) also studied CTCs and cfDNA from melanoma patients (37 patients). The researchers reported that although immunocytochemistry showed a vast heterogeneity of CTC morphology and phenotype, gene expression analysis via ddPCR of five melanoma-associated genes revealed a comparable trend in CTC and cfDNA scores. However, in some cases CTC analysis revealed changes in molecular signatures at the baseline and in post treatment that were complementary to ctDNA monitoring. Furthermore, this research describes the Parsortix^®^ yield as a suitable platform for potential downstream transcriptomic analysis due to its low white blood cell background yield as compared to other technologies [70].

## 5. Future Directions

As the omics revolution continues, we expect the further uptake of transcriptomics and proteomics to shape the future of liquid biopsies and personalised medicine for a comprehensive picture of tumour biology and clinical insights. The integration of multi-omics from laboratory bench to patient bedside faces challenges in translating vast and complex datasets into clinical benefit. Liquid biopsy-based multi-omics analysis is in its infancy, and standardisation and clinical feasibility are key to the successful integration of this tool into the clinic. This includes a need for improved access to microfluidic CTC isolation devices and sequencing platforms. However, the future of liquid biopsies is bright, with promising data emerging to support the use of whole blood as a source for multiple analytes providing information on disease prognosis, treatment selection, the monitoring of tumour evolution, and disease relapse. Furthermore, the use of dual analytes to discover complementary information will continue to emerge in the future literature, uncovering exclusive actionable insights to better inform personalised medicine. The future of the Parsortix^®^ system involves the development and commercialisation of a breadth of downstream assays to expand CTC analysis, via immunofluorescent and molecular solutions, to provide clinically actionable insight, as well as continued investigation into the dual analysis of CTCs and ctDNA.

## 6. Conclusions

Liquid biopsies are emerging as a less invasive, less costly, and safer tool that provide faster results and are more suited for longitudinal disease monitoring for cancer care. CTCs and ctDNA are described as cornerstones of liquid biopsy analysis, providing minimally invasive, real-time clinical information throughout the patient care pathway. Rapid advances in technology and the affordability of NGS continue to excel, paving the way for a new era of liquid biopsy. The omics revolution is driving the dual analysis of CTCs and ctDNA as complementary sources of genomic and transcriptomic information, as RNA emerges as a tool for more accurate phenotypical sampling. The Parsortix^®^ system is a versatile microfluidic device that facilitates epitope-independent capture and the analysis of CTCs in conjunction with the analysis of ctDNA from a single blood sample towards real-time personalised medicine, overcoming the shortfalls of immunoaffinity-based enrichment technologies that rely on epithelial surface markers known to understate CTC capture. In particular, the Parsortix^®^ system has been utilised in studies investigating the complementary information from CTCs and ctDNA for the evaluation of prognosis, to inform treatment selection and assess resistance and relapse, in some cases faster than the standard of care. This system addresses issues of sample heterogeneity and epitope-dependent CTC capture with label-free microfluidic isolation.

## Figures and Tables

**Table 1:** Publications using the Parsortix^®^ system: CTC and ctDNA analysis.

Study	Cancer	Patients	Analysis	Key Message	Reference
Markou et al. (2023)	Early-stage NSCLC	49	ddPCR of hotspot mutations *BRAF*, *KRAS*, *EGFR*, and *PIK3CA*	Dual analysis provided complementary molecular information and greater diversity in genomic information for cancer prognosis and treatment.	[62]
Markou et al. (2022)	Early-stage NSCLC	42	MSP of *APC*, *RASSFIA1*, *FOXA1*, *SLFN11*, *SHOX2*	Methylation profiles varied between CTCs, ctDNA, and primary tissue, suggesting that dual analysis allowed real-time monitoring of tumour evolution. A higher incidence of relapse was reported when at least one gene promoter is methylated in CTCs or cfDNA, highlighting the prognostic value of dual analysis.	[63]
Ntzifa et al. (2021)	NSCLC	42	DNA methylation patterns of *RASSF1A*, *RASSF10*, *APC*, *WIF-1, BRMS1*, *SLFN11*, *RARβ, SHISA3*, and *FOXA1*	CTCs and cfDNA provided complementary information and showed that methylation was associated with disease progression and identified as a potential resistance mechanism.	[64]
Ntzifa et al. (2021)	NSCLC	48	Crystal dPCR genotyping of EGFR mutations including *T790M*	Differences between ctDNA and CTCs show heterogeneity and could be predictive of resistance mechanisms useful for evolution tracking and treatment monitoring.	[66]
Ortolan et al. (2021)	TNBC	42	ddPCR and NGS, personalised panels including *TP53*, *PIK3CA*, *FGFR3* and more.	ctDNA and CTCs represent both spatial and temporal heterogeneity and allow dynamic monitoring of cancer progression.	[67]
Mondelo-Macia et al. (2020)	Lung, colon, prostate, melanoma, breast, and gastric	30	ddPCR for MET amplification in cfDNA and IF for MET overexpression in CTCs	CTC and cfDNA MET status analysis is a tool for monitoring resistance to anti-EGFR therapy and can inform overall survival.	[65]
Gorges et al. (2019)	Melanoma	84	Analysis of 61 clinically relevant variants across 13 genes including *BRAF*, *NRAS* and *MAP2K1*	Combined CTC and ctDNA analyses can reveal synergistic information, as well as predict relapse earlier than imaging and the standard of care in some cases.	[69]
Aya-Bonilla et al. (2020)	Melanoma	37	Immunocytochemistry of CTCs and ddPCR of *MLANA*, *TYR*, *MAGEA3*, *ABCB5* and *PAX3*	CTCs are a complementary feature to cfDNA monitoring and can be associated with shorter overall and progression-free survival.	[70]
